# Dynamic trajectory of multiple single-unit activity during working memory task in rats

**DOI:** 10.3389/fncom.2015.00117

**Published:** 2015-09-24

**Authors:** Xiaofan Zhang, Hu Yi, Wenwen Bai, Xin Tian

**Affiliations:** Department of Biomedical Engineering, School of Biomedical Engineering and Technology, Tianjin Medical UniversityTianjin, China

**Keywords:** rat, working memory, single unit activity, dynamic trajectory, maximal Lyapunov exponent

## Abstract

Working memory plays an important role in complex cognitive tasks. A popular theoretical view is that transient properties of neuronal dynamics underlie cognitive processing. The question raised here as to how the transient dynamics evolve in working memory. To address this issue, we investigated the multiple single-unit activity dynamics in rat medial prefrontal cortex (mPFC) during a Y-maze working memory task. The approach worked by reconstructing state space from delays of the original single-unit firing rate variables, which were further analyzed using kernel principal component analysis (KPCA). Then the neural trajectories were obtained to visualize the multiple single-unit activity. Furthermore, the maximal Lyapunov exponent (MLE) was calculated to quantitatively evaluate the neural trajectories during the working memory task. The results showed that the neuronal activity produced stable and reproducible neural trajectories in the correct trials while showed irregular trajectories in the incorrect trials, which may establish a link between the neurocognitive process and behavioral performance in working memory. The MLEs significantly increased during working memory in the correctly performed trials, indicating an increased divergence of the neural trajectories. In the incorrect trials, the MLEs were nearly zero and remained unchanged during the task. Taken together, the trial-specific neural trajectory provides an effective way to track the instantaneous state of the neuronal population during the working memory task and offers valuable insights into working memory function. The MLE describes the changes of neural dynamics in working memory and may reflect different neuronal population states in working memory.

## Introduction

Working memory refers to a cognitive system responsible for short-term mental storage and manipulation operations (Baddeley, 1992). Working memory appears to play a fundamental role in many high-level cognitive processes such as planning, reasoning, and decision-making. Prefrontal cortex (PFC) has been regarded as a brain structure closely linked to working memory (Funahashi and Kubota, 1994). Lesions or inactivation of the medial PFC (mPFC) in rodents lead to working memory impairments (Taylor et al., 2003; Yoon et al., 2008; Yang et al., 2014). Working memory can be modeled as dynamic patterns of neuronal population activity. Specifically, working memory has been proposed that is based on either a continuous attractor (Itskov et al., 2011) or a set of discrete attractors (Miller and Wang, 2006). In recent years, a popular theoretical view is that transient states rather than classical attractor states may better describe dynamical neural systems underlying sensation, perception, and cognition (Rabinovich et al., 2008; Buonomano and Maass, 2009). Transient states, as a basis for stimulus-response processes in neural systems, have been fruitfully applied in studies of the olfactory systems of locust (Stopfer et al., 2003; Mazor and Laurent, 2005; Broome et al., 2006) and zebrafish (Friedrich and Laurent, 2001), the visual system of turtle (Du et al., 2005), the statocysts of marine mollusk *Clione* (Levi et al., 2005). In these studies, neural trajectory, that is, the succession of transient states during neural processing was associated with different types of external stimuli, which serves as a new model for understanding neural encoding and decoding mechanisms in response to the external stimuli. Additionally, in content of cognition, the transient dynamics was also widely studied in the cognitive systems of motor planning (Shenoy et al., 2011; Churchland et al., 2012) and decision making (Balaguer-Ballester et al., 2011), which reveal important features of neural computation in cortical areas.

The common procedure to reveal neural trajectories involves a two-stage process: the single neuron spike trains are first smoothed over time and then a dimensionality reduction technique is performed (Yu et al., 2009). However, a state space constructed only from a few observations may not properly represent the geometry of neural trajectories of the underlying dynamical system. Therefore, Time delay embedding algorithm is primarily applied to the original population data in this work. To directly view the neural trajectory, methods that reduce the dimensionality of the data are typically needed. Principal components analysis (PCA) is one of the most commonly used data reduction methods in neuroscience. This method extracts orthogonal factors that best explain the variation in the data. Then a low dimensional space was constructed with a subset of the highest-variance components. In our work, kernel PCA (KPCA, a non-linear PCA) instead of PCA is used to project the delayed population data into a low dimensional state space. The main reason is that KPCA can take full advantage of the non-linear correlation between population vectors and effectively extract the non-linear features of the neuronal population activity (Lee et al., 2004). Generally, entropy (Caldirola et al., 2004) and complexity(Tononi et al., 1994) have been widely applied to describe the brain activity. In this work, the Lyapunov exponent is introduced to evaluate the dynamic characteristics of the neural trajectories. The method of Lyapunov exponents serves as a useful tool to quantify the exponential divergence or convergence of initially close nearby trajectories (Brown et al., 1991). It is common to refer to the largest one as the maximal Lyapunov exponent (MLE), which yields the greatest insight into the dynamics of the considered system. The MLE has proven its efficiency for the characterization of the EEGs during epileptic seizures (Chaovalitwongse et al., 2005; Nair et al., 2009) and schizophrenia (Kim et al., 2000).

In our work, we investigated the transient dynamics of the neuronal population activity in working memory. We simultaneously recorded the mPFC neurons with multiple-electrodes when rats performing a Y-maze working memory task. To gain insight into the spatio-temporal patterns of the neuronal population activity that represent working memory, time delay embedding theorems, and kernel principal components analysis (KPCA) were applied to multiple single-unit spike trains to reconstruct the neural trajectories in state space. The MLE was then selected to characterize the features of these neural trajectories in both correct and incorrect trials. The present study makes an attempt to reveal the underlying transient dynamics of working memory and the relationship with the behavioral performance of the animals.

## Materials and methods

### Ethics statement

Behavioral training and physiological experiments were conducted in accordance with the Care and Use of Laboratory Animals and approved by the Tianjin Medical University Animal Care and Use Committee.

### Apparatus and working memory task

**Figure 1:**
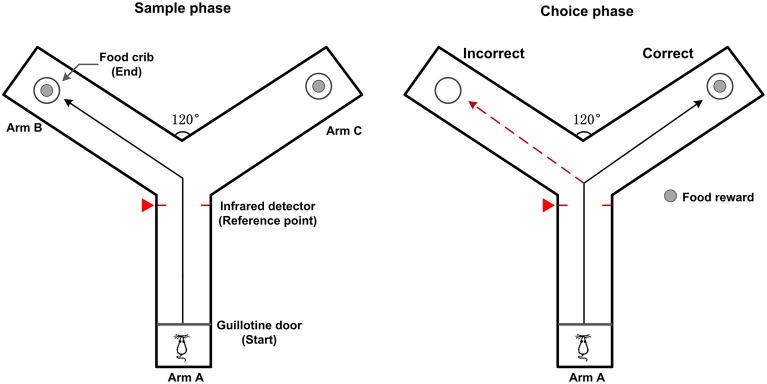
**Diagram of rat working memory task on a Y-maze**. The Y-maze apparatus have three identical plastic arms (same length) at a 120° angle from each other. A removable guillotine door is placed at the entrance and food pellets are placed in the food cribs at the end of the goal arms (Arm B and C). In the sample phase, the rat is placed at the start arm (Arm A). When the guillotine door is opened, the rat is free to enter either one of the goal arms to get a food reward. In the choice phase, the rat is rewarded for entering the arm that was not visited in the sample phase. The black solid line shows possible correct path, red dashed line shows possible incorrect path. The moments that the rat enters into the goal arm are detected by the infrared detector and marked by a red triangle (defined as the reference point).

Male Sprague-Dawley rats weighing 300–350 g were placed on a reverse light cycle upon arrival and given *ad libitum* access to water with food restriction (2 h a day to retain at least 85% of normal body weight) for two consecutive days. Then the rats were familiarized with a Y-maze for two days. After habituation, the rats received daily training sessions (10 trials per day) on a working memory (delayed alternation) task. The Y maze consists of three gray, opaque plastic arms (length × width × height: 75 × 14.5 × 15 cm), at a 120° angle from each other. One arm is designated as the “start” arm and the other two arms are assigned to be the “goal” arms (Figure 1). The working memory task was described as follows (Goldman et al., 1971). At the beginning of a trial, the rat was placed on the end of the start arm (arm A). Each trial included two phases: a sample phase and a choice phase. In the sample phase, the rat could get some food in the crib as a reward when it arrived at the end of the goal arm (arm B or arm C). Then the rat returned to the start arm to make a free choice after a 5 s delay. In this choice phase, the rat received a food reward only if it entered the arm not visited in the sample phase. Consecutive visit to the same goal arm was defined as an error. Between trials, the arms were wiped with alcohol to remove potential olfactory cues. The occurrence of behavioral events indicating the rat turned into the goal arm was marked by an infrared sensor in the Y-maze and the corresponding time stamp was defined as the reference point.

After reaching the performance criterion (at least 80% correct out of the total trials in two consecutive days), the rat received a chronic implant surgery under aseptic conditions. The rat was anesthetized with sodium pentobarbital (40 mg/kg) and a 16-channel micro-electrode array (nickel-chromium, < 1 MΩ) was implanted into the mPFC (see Bai et al., 2012) for an in-depth description of the surgical procedures). The array contained 2 × 8 electrodes (2 × 0.3 mm^2^ in area, 33 μm in diameter, inter-electrode space of 200 μm, made in house). According to the rat brain stereotaxic coordinates, the microelectrode array was implanted into the PL cortex of the mPFC (2.5–4.5 mm anterior to bregma, 0.2–1.0 mm lateral to midline, 2.5–3.5 mm deep from cortical surface). After the surgery, the rats were allowed to recover for 7 days.

After recovery, multi-channel neuronal activity was recorded *in vivo* with a Cerebus Acquisition System (Cyberkinetics, USA) when the rats performing the task. The whole time course of the working memory task was captured by EthoVision XT video tracking system (Noldus, USA).

### Data preprocessing

The recordings were sampled at 30 kHz, filtered from 250–7500 Hz and stored off-line with time stamps in the Neural Signal Processor. Spikes were detected using a preset voltage threshold. Then spike sorting was performed using Offline Sorter (Plexon Inc, USA) to separate out the single-unit activity. Single neurons with a very low baseline firing rate (< 30 spikes/min) or a signal-to noise of < 3.0 were discarded.

### Neural trajectories reconstruction

The spike trains were first convolved with Gaussian kernels to obtain the instantaneous firing rates (bandwidth: 200 ms). For a single trial, the smoothed firing rates of the simultaneously recorded neurons can be defined as population vectors, embedded within a multidimensional state space:

(1) $$X={\left[x_{1}(t),x_{2}(t),\cdots ,x_{N}(t)\right]}^{T}$$

where *N* is the number of neurons. Thus, a single point (e.g., the current firing rates of all recorded neurons) in this space represented the whole state of the recorded neuronal population at a given time. Over the course of the working memory task, these points formed a neural trajectory through state space, beginning, and ending at a point or a number of points that represented baseline activity. Then, time delay embedding algorithm was primarily applied to the original population vectors. The method is that the dimensionality of the state space can be expanded by adding time-lagged vectors of the original data as new variables to the space. The delayed vector can be constructed as follow:

(2) $$Y={\left[x_{i}(t),x_{i-\tau }(t),\cdots ,x_{i-(m-1)\tau }(t)\right]}^{T},i=\text{1},\text{2},\cdots ,N$$

where *m* refers to the embedding dimension and τ refers to the time lag. The false nearest neighbor method was used to determine the minimal sufficient embedding dimension (Kennel et al., 1992). The optimal time lag can be estimated with the help of the time delayed mutual information (Fraser and Swinney, 1986).

Sequentially, KPCA was used to project the delayed population vector into a low dimensional state space (Schölkopf et al., 1998). The principal components (PCs) were acquired and organized in descending order of their variance. Then, the neural trajectory was constructed by the PCs, with each dimension representing the individual PCs.

### Quantitative description of neural trajectory

For a *n*-dimensional dynamical system, it has *n* Lyapunov exponents λ_*i*_(*i* = 1, 2, ···, *n*), which describe the divergence or convergence of nearby trajectories traced out during system evolution in the state space. The system is defined to be chaotic if at least one of the Lyapunov exponents is positive and the initially close points will diverge to any arbitrary separation. The first Lyapunov exponent was termed as maximum Lyapunov exponent (MLE). In this study, the MLE was calculated by Wolf's algorithm (Wolf et al., 1985). The MLE can be presented as:

(3) $$MLE=\frac{1}{t_{M}-t_{0}}\sum_{k=\text{1}}^{M}log_{2}\frac{L^{\prime }(t_{k})}{L(t_{k-1})}$$

where *L*(*t*_*k*−1_) is the Euclidean distance at the time point *t*_*k*−1_ of two nearby trajectories and at the next time point *t*_*k*_. The distance between the same two trajectories can evolve to length *L*′(*t_k_*). *M* is the number of local MLE estimations computed within the time period *T* of the data segment, where *T* = *t*_*M*_ − *t*_0_.

### Statistical analysis

In this work, we described 40 correct trials and 32 incorrect trials from four rats, with 10 correct trials and eight incorrect trials from each rat. All the data are expressed as mean ± SEM. The paired *t*-test was performed to test the significant differences of neural trajectories during working memory and under resting state. Besides, the independent-samples *t*-test was used to evaluate the significant differences of neural trajectories between the correct and incorrect trials. For all comparisons, the statistical significance levels were set at different levels: ^*^P < 0.05, ^**^P < 0.01, ^***^P < 0.001.

## Results

### Neuronal population activity during working memory task

**Figure 2:**
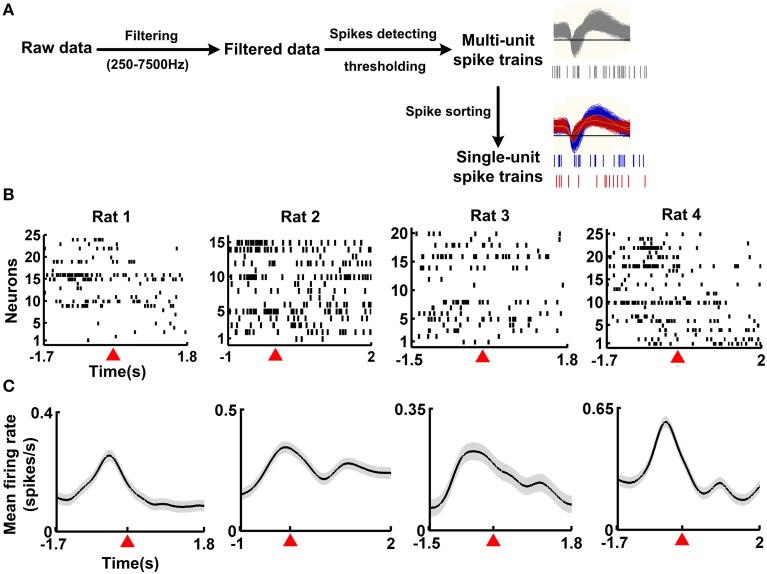
**Single-unit activity from four example correct trials during the working memory task**. **(A)** Spike detection and sorting from the recordings. The raw data was filtered (250–7500 Hz) and multi-unit spikes were detected using a preset threshold. Then, single-unit spike trains were isolated from the multi-unit spikes. **(B)** Raster plots of neuronal activity during the working memory task for 4 rats. For each rat, one correct trial was shown. Each row plotted the response of an individual neuron. Time 0 represented the reference point, marked by the red triangle. **(C)** Mean firing rates of neuronal population during the working memory task. Data from **(B)** were used, with 10 correct trials for each rat. Shaded areas indicated the SEM. Time 0 represents the reference point, marked by the red triangle.

Spike trains of single neurons were obtained (Figure 2A) from 40 correct trials (four rats, 10 correct trials for each rat). The time course of the working memory task was 3.5 ± 0.21, 3 ± 0.19, 3.3 ± 0.15, 3.7 ± 0.23 s (mean ± SEM) for rat 1, rat 2, rat 3, and rat 4, respectively. Besides, for each rat, the resting-state activity was taken during the inter-trial intervals. A typical example shows the changes in neuronal population activity during the working memory task (Figure 2). The neuronal population firing increased rapidly prior to the reference point and decayed down to baseline level after the reference point (Figure 2B). Then the spike trains were transformed into smooth, continuous-time firing rates with a 200-ms Gaussian kernel. The neuronal population activity profile was shown in Figure 2C. The neuronal population firing rates increased, peaked around 500 ms prior to the reference point and then declined to baseline.

### Dynamic trajectories of working memory in state space

**Figure 3:**
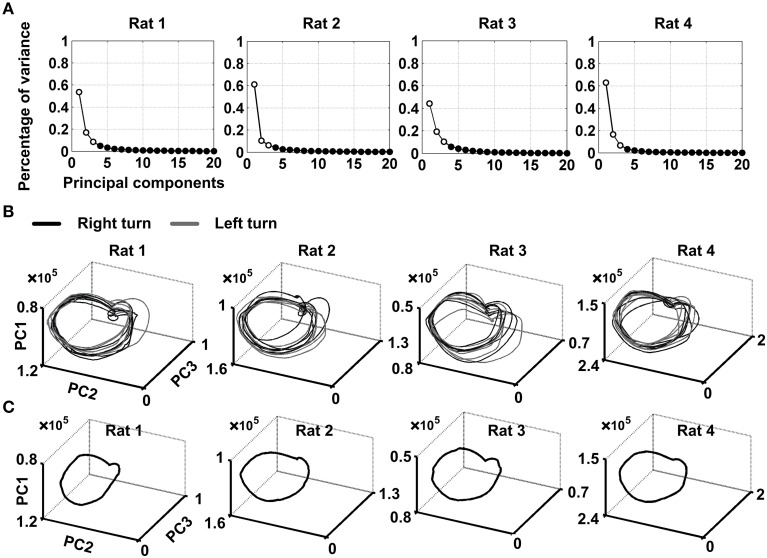
**Visualization of neural trajectories representing multiple single-unit activity over time**. **(A)** Scree plot of the principal components (PCs) obtained from the smoothed firing rate. The PCs were calculated over 10 correct trials for each rat via KPCA. Time slice points calculated from neuronal spike activity of four rats, projected onto state space using the first three PCs. The minimum embedding dimension *m* = 3 and the optimal time delay τ = 200 ms were applied for time-delay embedding. **(B)** Shown were 10 individual-trial neural trajectories for each rat. Gray arrows indicated the direction of evolution of neural trajectories during working memory. **(C)** Trajectory averaged over 10 trials for each rat.

The minimum embedding dimension *m* = 3 and the optimal time delay τ = 200 ms were chosen for time-delay embedding. The cumulative variance explained by the first three PCs is above 70% (79 ± 2, 78 ± 4, 74 ± 1, and 86 ± 2% for the four rats, mean ± SEM) (Figure 3A). As the first three PCs capture the most of the variance, it is appropriate to transform the original data to a low dimensional space defined by the first three PCs to reveal the transient dynamics as neural trajectory. The individual trajectories and averaged trajectory during the working memory task were shown in Figures 3B,C. Each point along the trajectory represented the instantaneous neuronal population activity during working memory. It can be seen quite clearly from the Figure 3B that the trajectories were quite similar on the correct trials across correct trials for four rats. Besides, the trajectories for correct right-turn trials and left-turn trials evolved in a similar way. This suggested that the trajectories contain specific information dependent on neuronal activity during working memory. In this view, a neuronal sequence of activity may be activated during working memory task. The neuronal activity may exhibit different dynamics over time and formed a trajectory in state space.

### Maximal lyapunov exponent of neural trajectory

**Figure 4:**
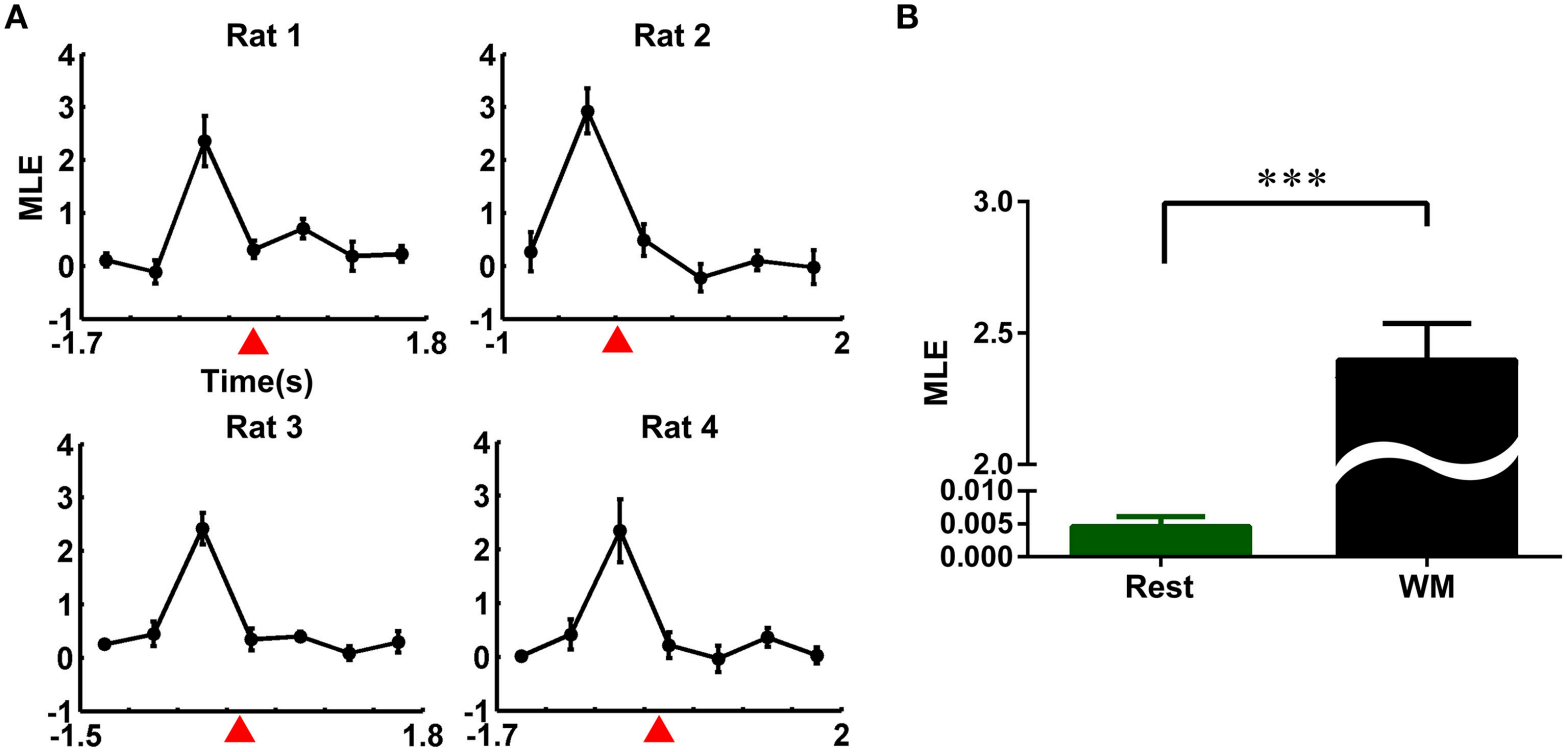
**Evolution of the maximal Lyapunov exponent (MLE) of neural trajectories during the working memory task**. **(A)** Changes of the MLEs during the working memory task in the correct trials (averaged over 10 trials for each rat). **(B)** MLEs in working memory and resting state. The MLEs in working memory state were significantly higher than those in resting state (averaged over 40 correct trials from four rats, ^***^P < 0.001).

To quantify these qualitative observations from a dynamics perspective, we calculated the MLE for four rats. The whole time course of the working memory task and resting state were divided into non-overlapping 500 ms time bins, respectively. The MLEs during the working memory task were calculated in each time bin, combining the individual trajectories with the averaged trajectory. The results were shown in Figure 4. The MLEs changed consistently across the four rats, increased obviously and peaked prior to the reference point, and followed by a steady decline (Figure 4A). Then, we compared the neuronal activity at working memory state (time duration: pre-0.5 s and post-0.5 s the peak of the MLEs) with that at the resting-state (40 correct trials from four rats, mean ± SEM, Figure 4B). The MLEs during working memory were significantly higher than those in the resting-state (2.3970 ± 0.1394 vs. 0.0051 ± 0.0003, ^***^P < 0.001).

**Figure 5:**
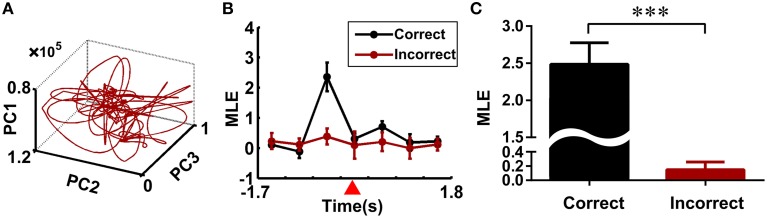
**Neural dynamics of the multiple single-unit activity in the incorrect trials**. **(A)** Neural trajectories of the multiple single-unit activity in the incorrect trials (eight incorrect trials from rat 1 were analyzed). **(B)** Changes of the MLEs during the working memory task in the correct and incorrect trials. The reference point was marked by a red triangle. **(C)** Comparison of the MLEs between the correct and incorrect trials. The peak of the MLEs in the correct trials was significantly higher than those in the incorrect ones (^***^P < 0.001).

**Figure 6:**
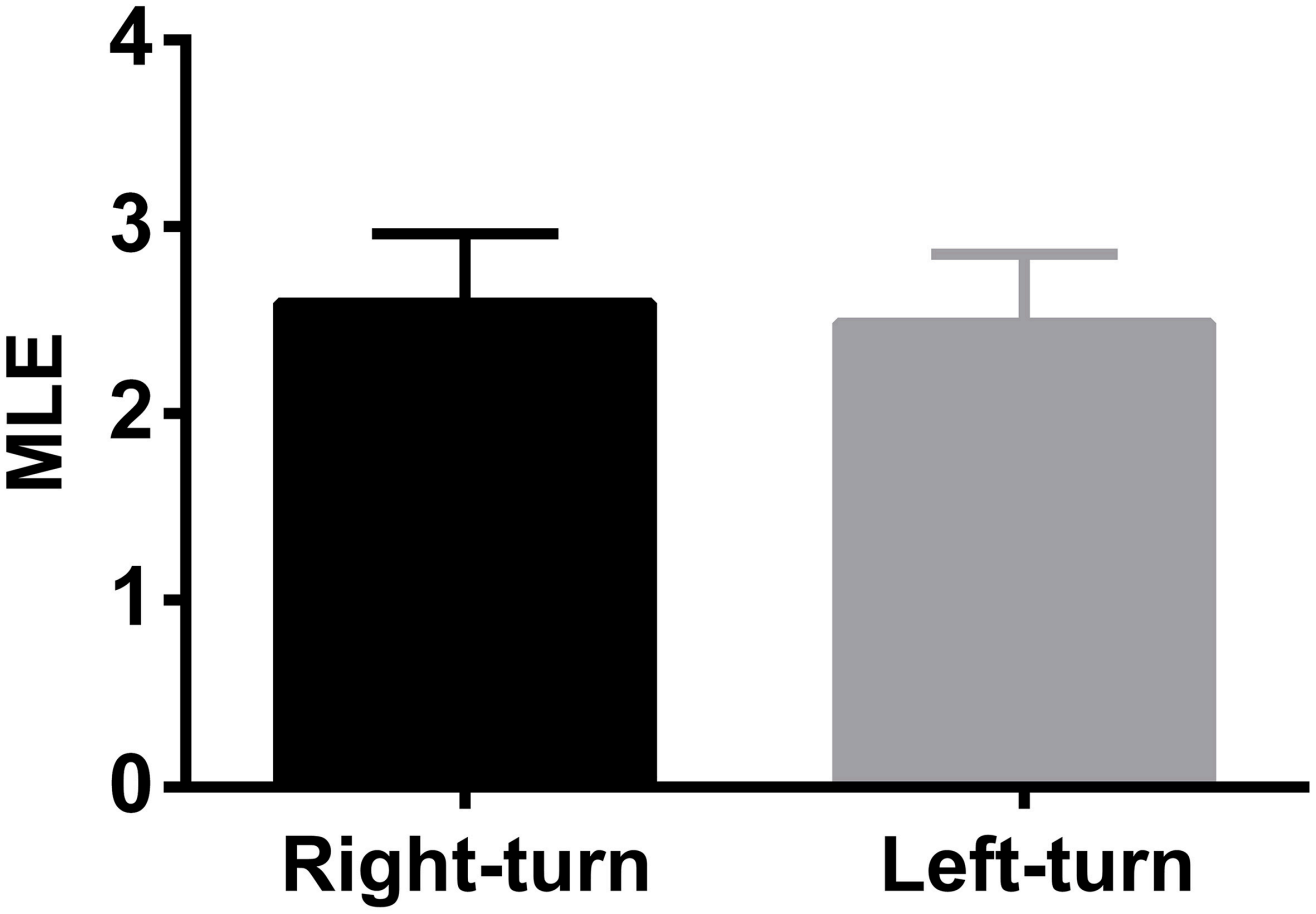
**The comparison of MLEs of dynamic trajectories for 20 correct right-turn trials and for 20 left-turn trials**. There was no significant difference for the two correct cases (*P* > 0.05).

To test whether the trajectory pattern and MLE were significant relative to working memory dynamics. We constructed the trajectories and calculated the corresponding MLE for incorrect trials. Neural trajectories over eight incorrect trials (from rat 1) were shown in Figure 5A. Accordingly, the changes of the MLEs in the incorrect trials were shown in Figure 5B. The neural trajectories during the same duration (duration: the time bin corresponding to the peak of the MLEs) in the correct and incorrect trials were further compared (Figure 5C). The peak of the MLEs in the correct trials was significantly higher than those in the incorrect trials (2.4860 ± 0.2882 vs. 0.1486 ± 0.1095, ^***^P < 0.001). Furthermore, the question arises here, whether the dynamics of trajectories have any difference for correct right-turn trials and left-turn trials? To answer the above question, we calculated the MLE as a qualitative description for trajectory dynamics, the MLEs were compared for the above two conditions, the results were shown in Figure 6, there was no significant difference (2.5092 ± 0.3739 vs. 2.4786 ± 0.3683, *P* > 0.05).

From the results mentioned above, it was suggested that the action potentials encode the information in working memory, and the trajectory pattern decode the performance of working memory.

## Discussion

This study explored the neural trajectories of the neuronal population activity in working memory. We combined the state space reconstruction methods with KPCA algorithm to extract the neural trajectory and employed the MLE to characterize this transient property of neuronal population activity. The conclusions of this work are as follows: the stable and transient nature of the neural population was presented in the state space corresponding to the correct trials during the working memory task; the MLEs increased and peaked before the rats arrived at the choice point and then declined to baseline in the correct trials, reflecting the dynamical feature of the neural trajectories.

Previous study has established the direct links between neural transient activity and sensory or environmental representations with the aid of neural trajectories. Stopfer et al. (2003) found that odor identity and intensity could be determined by the neural trajectories reconstructed from the firing pattern of a population of antennal lobe projection neurons in the locust. In this work, the neural trajectories are reproducible and robust across different correct trials (Figure 3B). Trajectories were highly variable on incorrect trials (Figure 5A). Among all the trials, we can easily recognize the incorrect ones, whose neural trajectories appeared different from those in the correct trials.

With the assistance of MLE, the dynamical changes of the neural trajectories were revealed. The neuronal population activity may dynamically evolve on different intrinsic states, considering the significant variation of the MLEs during the working memory task. Balaguer-Ballester et al. (2011) revealed the several dynamic states associated with decision making task. The different states toward which the neuronal population activity evolves corresponding to different cognitively defined task stages, such as training phases, delay phases relate to working memory load, choices the rat makes, and rewards it receives.

In summary, we investigated the transient dynamics of neuronal population in working memory. The neural trajectory provides concise descriptions of working memory function and can be used for neural decoding of the performance of working memory. Transient dynamics may therefore be a common framework for working memory, as were demonstrated in neural functions related to memory such as motor planning (Churchland et al., 2012), decision making (Harvey et al., 2012). Besides, the MLE may offer an effective way to describe the characteristics of neural dynamics during working memory. Furthermore, these findings may provide a support for investigating the neural dynamics of working memory in the disease model of rats or mice.

### Conflict of interest statement

The authors declare that the research was conducted in the absence of any commercial or financial relationships that could be construed as a potential conflict of interest.
